# Prevalence of Sleep Disturbances and Sleep Quality in Chinese Healthcare Workers During the COVID-19 Pandemic: A Systematic Review and Meta-Analysis

**DOI:** 10.3389/fpsyt.2021.646342

**Published:** 2021-02-18

**Authors:** Lei Xia, Changhao Chen, Zhiqiang Liu, Xiangfen Luo, Chunyan Guo, Zhiwei Liu, Kai Zhang, Huanzhong Liu

**Affiliations:** ^1^Department of Psychiatry, Chaohu Hospital of Anhui Medical University, Hefei, China; ^2^Department of Psychiatry, Suzhou Second People's Hospital, Suzhou, China; ^3^Department of General Medicine, Chaohu Hospital of Anhui Medical University, Hefei, China; ^4^Department of Psychiatry, The Second Affiliated Hospital of Bengbu Medical College, Bengbu, China; ^5^Department of Psychiatry, Fuyang Third People's Hospital, Fuyang, China

**Keywords:** COVID-19, healthcare workers, sleep disturbances, China, meta-analysis

## Abstract

**Objectives:** Healthcare workers (HWs) experienced high levels of work stress during the COVID-19 pandemic, leading to a high risk of sleep disturbances. This meta-analysis aimed to explore the prevalence of sleep disturbances and sleep quality in Chinese HWs during the COVID-19 pandemic.

**Methods:** English (PubMed, EMBASE, PsycINFO, Web of Science, and the Cochrane Library) and Chinese databases (WanFang, Chinese National Knowledge Infrastructure, and SinoMed) were systematically and independently searched for relevant studies published from December 1, 2019, to May 20, 2020. The pooled prevalence of sleep disturbances and sleep quality were calculated using a random-effects model.

**Results:** A total of 17 studies involving 12,682 Chinese HWs were included in the meta-analysis. The pooled prevalence of sleep disturbances in Chinese HWs was 45.1% (95% CI: 37.2–53.1%). We found that the prevalence of sleep disturbances varied among frontline, infected, and non-frontline HWs (*Q* = 96.96, *p* < 0.001); females and males (*Q* = 9.10, *p* = 0.003); studies using different assessment instruments (*Q* = 96.05, *p* < 0.001); and studies with different sample sizes (*Q* = 5.77, *p* = 0.016) and cut-off values (*Q* = 62.28, *p* < 0.001). The pooled mean total score of the Pittsburgh Sleep Quality Index (PSQI) was 9.83 (95% CI: 8.61–11.04). HWs in Wuhan had a higher total PSQI score than those in other regions (*Q* = 9.21, *p* = 0.002).

**Conclusion:** Sleep disturbances were common in Chinese HWs during the COVID-19 pandemic, particularly in frontline and infected HWs. Our results indicate the heavy mental health burden on HWs during the COVID-19 pandemic in China and can provide other countries with valuable information to assist HWs during the crisis.

## Introduction

In December 2019, the 2019 novel coronavirus disease (COVID-19) was officially reported for the first time in Wuhan, Hubei Province, China; it then spread rapidly worldwide (1). Patients infected with COVID-19 may develop acute respiratory distress syndrome, which is associated with a high risk of intensive care unit (ICU) admission and mortality (2). By the end of May 2020, more than 84,000 people in China had been confirmed to have COVID-19 (3).

In recent months, people have been directed to stay at home to minimize the spread of COVID-19; however, healthcare workers (HWs) are at high risk of infection due to the nature of their work in fighting the virus. According to official statistics, a total of 3,387 Chinese HWs were infected with COVID-19 during the COVID-19 pandemic, and more than 90% of them were from Hubei (4). As the number of cases in China increased, HWs had to care for an increasing number of confirmed and suspected cases that required strict isolation. Due heavy workloads, HWs tended to experience excessive fatigue, tension, and even exhaustion (5). They were worried about themselves and their families being infected, and they were also concerned about their family members' worrying about them. In addition, they may have become overexcited in clinical work and refused reasonable rest to ensure their health (6).

A previous study in Toronto reported that 29% of HWs suffered from emotional distress during the acute respiratory syndrome (SARS) outbreak in 2003 (7). Another study in Hong Kong found that 68% of frontline HWs experienced high levels of stress, and 57% reported psychological distress (8). COVID-19 has a stronger ability to spread than SARS (9). The COVID-19 pandemic was more panic-inducing than the SARS epidemic and created new challenges regarding the mental health of key population subgroups, such as HWs. HWs experienced high levels of work stress during the COVID-19 pandemic, which may have led to an increased risk of sleep disturbances (10, 11).

Poor sleep can weaken HWs' attention and decision-making ability and reduce clinical work efficiency (12), which may hinder the fight against COVID-19. Moreover, sleep disturbances can contribute to the development of many other mental health problems in HWs, such as depression and anxiety (13–15), and may have an impact on their long-term health (16). Undoubtedly, sleep disturbances are very harmful to HWs and deserve our attention. If HWs have early signs of mental health problems, such as poor sleep quality or insomnia complaints, early interventions should be performed to minimize the risk of mental illness.

The prevalence of sleep disturbances in Chinese HWs during the COVID-19 pandemic varies greatly among studies, ranging from 11.3 to 100% (17, 18). To date, there is no meta-analysis of the prevalence of sleep disturbances and sleep quality in Chinese HWs during the COVID-19 pandemic despite the fact that many relevant studies have been published in Chinese. Thus, we conducted this meta-analysis to explore the prevalence of sleep disturbances and sleep quality in Chinese HWs during the COVID-19 pandemic using English- and Chinese-language databases and the possible reasons for the inconsistencies in the current estimates.

## Methods

### Search Strategy

This study was conducted and reported in accordance with the Preferred Reporting Items for Systematic Reviews and Meta-Analyses (PRISMA) guidelines (19) and the Meta-analyses Of Observational Studies in Epidemiology (MOOSE) recommendations (20). The study was registered in PROSPERO under registration number CRD42020186785.

Two independent investigators (LX and Z-QL) systematically searched English databases (i.e., PubMed, Embase, PsycINFO, Web of Science, and the Cochrane Library) and Chinese databases (i.e., WanFang, Chinese National Knowledge Infrastructure [CNKI], and SinoMed) for articles published from December 1, 2019, to May 20, 2020, using the following terms: (“sleep disorder” OR “sleep disturbance” OR “insomnia” OR “sleep problem” OR “sleep symptom” OR “sleep quality”) AND (“epidemiology” OR “cross-sectional study” OR “prevalence”) AND (“COVID-19” OR “SARS-CoV-2” OR “2019-nCoV” OR “SARS-CoV-2”) AND (“health personnel” OR “medical staff” OR “healthcare workers”). An additional search was also performed manually by inspecting the references of the identified studies.

### Study Selection

Studies were included if they fulfilled the following criteria: (a) the subjects were HWs, including doctors, nurses, and other HWs (such as hospital administration staff), who worked in hospitals or other medical institutes; (b) the studies had cross-sectional or cohort study designs (we extracted only the baseline data) and conducted in China during the COVID-19 pandemic; (c) the studies included data on the prevalence of sleep disturbances as measured by sleep questionnaires (such as the Pittsburgh Sleep Quality Index [PSQI], Insomnia Severity Index [ISI], and Athens insomnia scale [AIS]) or standard diagnostic criteria, and/or sleep quality was measured by the PSQI with means and standard deviations reported. Of note, studies with unclear assessment instruments or studies that reported neither the prevalence of sleep disturbances nor the mean score of sleep quality were excluded. Case studies, reviews, and duplicate articles were also excluded. Two investigators (LX and Z-QL) screened the potentially relevant studies independently. Any disagreement during the study selection process was resolved through discussion with a third investigator (HL).

### Data Extraction and Quality Assessment

Two investigators (LX and Z-QL) conducted data extraction independently, and a third investigator (HL) checked the final data. The following information was extracted from each identified study: first author, publication year, geographic location, study time (when each study was conducted), sampling methods, sample size, study design, proportion of HWs in Wuhan, proportion of frontline HWs, proportion of infected HWs, proportion of female HWs, mean age, assessment instruments, cut-off scores for sleep disturbances, number of HWs with sleep disturbances, and mean score of sleep quality. During data extraction, frontline HWs were considered to be HWs who were currently working in fever clinics and designated isolation hospitals or wards to care for confirmed and suspected COVID-19 patients (21). Infected HWs were those HWs who had been diagnosed with COVID-19 before the investigation began and were not included in frontline or non-frontline HW groups. In this study, the main outcome was the prevalence of sleep disturbances and the secondary outcome was sleep quality measured by the PSQI, with means and standard deviations reported. We evaluated the quality of each included study using an 8-item assessment tool for epidemiological studies, with a total score of 0–8 (high quality: ≥7; moderate quality: 4–6; and low quality: ≤ 3) (22, 23).

### Statistical Analysis

We conducted data synthesis and analysis using STATA version 12.0 (Stata Corporation, College Station, TX, USA) and Comprehensive Meta-Analysis Version 2 (Biostat Inc., Englewood, NJ, USA). As in previous studies (24, 25), the random-effects model was used to calculate the pooled prevalence of sleep disturbances and the mean total score of the PSQI with 95% confidence intervals (CIs). The *I*^2^ statistic was used to estimate the studies' heterogeneity, with *I*^2^ > 50% indicating significant heterogeneity (26). Subgroup analyses of pooled prevalence were performed considering the following variables: assessment instrument, sex, area (Wuhan/other regions), type of HWs (frontline/non-frontline/infected HWs), professional group (doctors/nurses), sample size (<927 or ≥927), and PSQI cut-off values (≥7 or ≥7). If more than 10 studies were included, meta-regression analyses were performed to detect heterogeneity in continuous variables (i.e., sample size, mean age, and study quality) (27). Publication bias was evaluated with funnel plots and Egger's test (28). The “trim and fill” method was used to estimate the number of missing studies using a random-effects model with a “linear” estimator. Sensitivity analysis was conducted by subtracting each study sequentially and evaluating the consistency of the results to identify studies that may affect the prevalence and the total scores. Statistical significance was set at a *P*-value of 0.05 (two-tailed).

## Results

### Search Results

**Figure 1:**
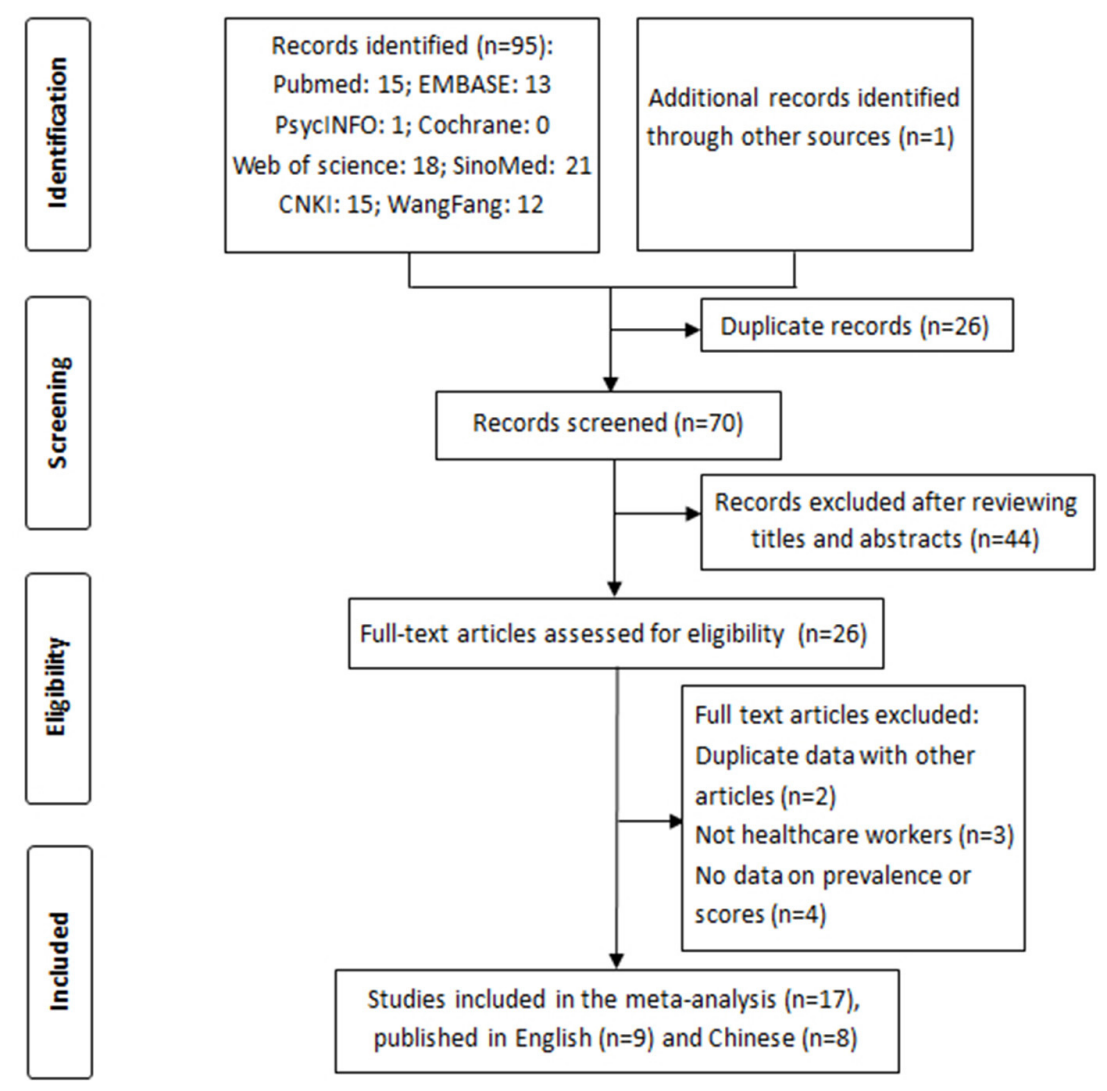
Flowchart of the study selection process.

A total of 96 potentially relevant studies were initially identified. After removing duplicate records, the titles and abstracts of 70 studies were screened. After full-text reviews of the remaining 26 studies, nine studies were excluded due to the following reasons: duplicate data (*n* = 2); non-HWs study population (*n* = 3); and missing prevalence data or scores (*n* = 4). Finally, seventeen studies (17, 18, 21, 29–42) (nine published in English and eight published in Chinese) were included (Figure 1).

### Study Characteristics and Quality Assessment

Table 1 lists the general characteristics of the 17 included studies involving 12,682 Chinese HWs (sample size range, 92–2,250). Of note, these studies were conducted across China during the implementation of closed-off management that started in January 2020 [Wuhan reopened on April 8 after a 76-day lockdown (43)]. The mean age of the HWs ranged from 29.0 to 43.2 years, and the proportion of females ranged from 55.1 to 98.3%. Most studies (64.7%, 11/17) used the PSQI; four studies used the ISI, two used the AIS, and one used four items from the PSQI. In total, 88.2% (15/17) of the studies reported the prevalence of sleep disturbances, and 52.9% (9/17) reported mean sleep quality scores with standard deviations. The mean quality assessment score was 4.8 (range, 4–6), and all the included studies were rated as having “moderate quality” (Supplementary Table 1).

**Table 1:** Characteristics of the studies included in the meta-analysis.

**No**.	**Principal author (year)**	**City/Province; Time**	**Sampling method**	**Sample size**	**Study design**	**HWs in Wuhan (%)**	**Frontline HWs (%)**	**Infected HWs (%)**	**Mean age (mean±SD)**	**Female (%)**	**Sleep information**	**Instrument**	**Cut-off**	**Quality score**
1	Li X 2020	Wuhan, Ningbo; 15/02–22/03	O	948	Cross- sectional	23.1	23.1	0	NA	76.8	Insomnia (%)	AIS	≥6	5
2	Zhou Y 2020	Liaoning, Hubei; 21/02–06/03	O	1,001	Cross- sectional	NA	100	0	33.8 ± 6.6	88.9	Insomnia (%)	PSQI	≥8	5
3	Zhang C 2020	Hubei and other regions; 29/01–03/02	O	1,563	Cross- sectional	NA	44.1	0	NA	82.7	Insomnia (%)	ISI	≥8	4
4	Zhang W 2020	NA; 19/02–06/03	O	927	Cross- sectional	NA	14.9	0	NA	73.1	Insomnia (%)	ISI	≥8	4
5	Lai J 2020	Wuhan, Hubei, and other regions; 29/01–03/02	CL, R	1,257	Cross- sectional	60.5	41.5	0	NA	76.7	Insomnia (%)	ISI	≥8	6
6	Yin Q 2020	NA; 01/02–05/02	O, SN	371	Cross- sectional	3.2	34	0	35.30 ± 9.48	61.5	Insomnia (%)	Four questions from PSQI	≥3	5
7	Wang S 2020	Wuhan; 30/01–07/02	O	123	Cross- sectional	80.5	0	0	33.75 ± 8.41	90.2	PSQI score (M ± SD, %)	PSQI	≥8	4
8	Huang Y 2020	NA; 03/02–17/02	O	2,250	Cross- sectional	NA	NA	0	NA	NA	Insomnia (%)	PSQI	≥8	5
9	Wu K 2020	Wuhu; Before 07/03	O	120	Cross- sectional	0	50	0	33.5 ± 12.4, 33.8 ± 11.9^a^	74.1	PSQI score (M ± SD, %)	PSQI	≥7	5
														
10	Deng L 2020	Zhongshan; Before 10/03	NA	230	Cross- sectional	0	100	0	NA	96.7	PSQI score (M ± SD)	PSQI	NA	5
11	He Y 2020	Wuhan; 24/01–02/03	O	256	Cross- sectional	100	85.2	14.8	38.7 ± 6.3, 43.2 ± 7.2^b^	55.1	PSQI score (M ± SD, %)	PSQI	≥8	4
12	Liu X 2020	Bejing; 01/02–18/02	O	1,097	Cross- sectional	0	NA	0	29.00 ± 5.88	98.3	Insomnia (%)	ISI	≥8	5
13	Mei J 2020	Wuhan; Before 09/02	O	140	Cross- sectional	100	NA	50.0	35.10 ± 8.39 36.24 ± 9.20^c^	77.1	PSQI score (M ± SD, %)^d^	PSQI	≥7	4
14	Nong Q 2020	Nanning; 31/01–03/02	O, CL, R	92	Cross- sectional	0	0	0	32.60 ± 5.07	68.5	PSQI score (M ± SD, %)	PSQI	≥8	5
15	Wei L 2020	Shanghai; Before 16/03	O, CL, R	2,150	Cross- sectional	0	0	0	NA	59.4	PSQI score (M ± SD, %)	PSQI	≥8	6
16	Wu J 2020	Sichuan; Before 02/02	O, CO	106	Cross- sectional	0	100	0	30.84 ± 4.52	80.2	PSQI score (M ± SD, %)	PSQI	≥8	5
17	Li X 2020	Wuhan; Before 24/03	O	121	Cohort	100	100	0	30.32 ± 5.39	89.3	PSQI score (M ± SD)	PSQI	NA	4

*HWs, healthcare workers; NA, not applicable*.

*Instruments: PSQI, Pittsburgh Sleep Quality Assessment; ISI, Insomnia Severity Index; AIS, Athens insomnia scale*.

*Mean age (years), the mean age of all HWs in each study, except those listed below*:

a *33.5 ± 12.4 and 33.8 ± 11.9 in frontline (n = 60) and non-frontline HWs (n = 60), respectively*;

b *38.7 ± 6.3 and 43.2 ± 7.2 in frontline (n = 218) and infected HWs (n = 38), respectively*;

c *36.24 ± 9.20 and 35.10 ± 8.39 in infected (n = 70) and non-infected HWs (n = 70)*.

d *This study reported only the prevalence of sleep disturbances in infected HWs (n = 70)*.

*Sampling methods: O, online; CL, cluster sampling; CO, convenient sampling; R, random sampling; SN, snowball sampling; ST, stratified sampling*.

*Frontline HWs were considered to be HWs who were currently working in fever clinics and designated isolation hospitals or wards to care for confirmed and suspected COVID-19 patients. Infected HWs were those HWs who had been diagnosed with COVID-19 before the investigation began*.

### Prevalence of Sleep Disturbances

**Figure 2:**
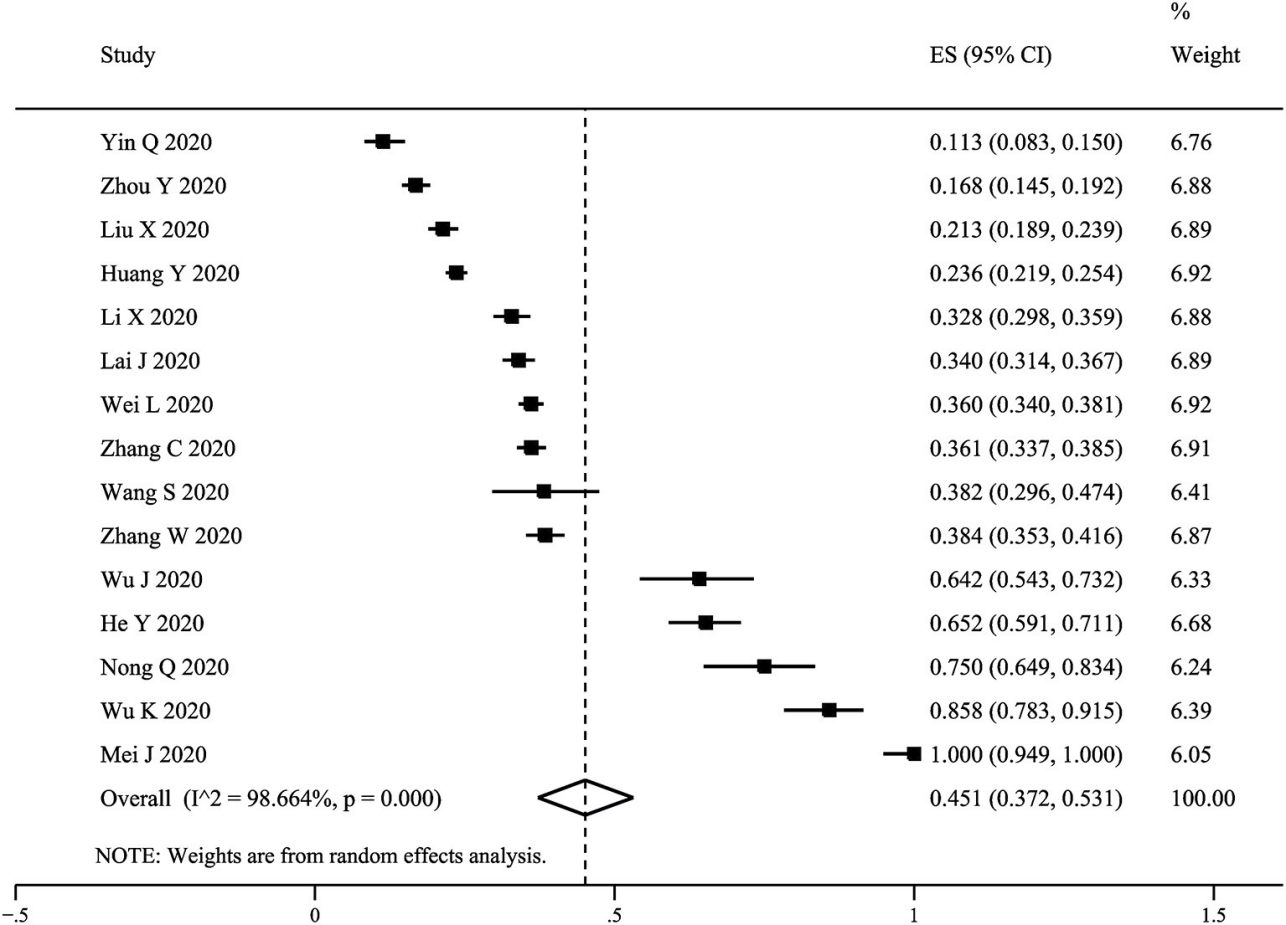
Forest plot of the prevalence of sleep disturbances in Chinese healthcare workers.

The pooled prevalence of sleep disturbances in 15 studies with 12,261 HWs was 45.1% (95% CI: 37.2–53.1%, *I*^2^ = 98.7%) (Figure 2). Table 2 presents the results of the subgroup analyses of the prevalence of sleep disturbances in Chinese HWs. There was a significant difference in prevalence among studies using different assessment instruments, at 58.0% (95% CI: 43.4–71.9%) in those that used the PSQI, 32.2% (95% CI: 25.1–39.9%) in those that used the ISI, 32.8% (95% CI: 29.8–35.9%) in those that used the AIS, and 11.3% (95% CI: 8.3–15.0%) in the study that used four items from the PSQI (*Q* = 96.05, *p* < 0.001). The prevalence rates of sleep disturbances among frontline, infected, and non-frontline HWs were 57.4% (95% CI: 38.8–75.0%), 97.0% (95% CI: 92.5–99.7%), and 40.0% (95% CI: 29.9–50.6%) (*Q* = 96.96, *p* < 0.001), respectively. The prevalence of sleep disturbances in females (36.7%, 95% CI: 34.8–38.7%) was higher than that in males (28.6%, 95% CI: 24.2–33.2%) (*Q* = 9.10, *p* = 0.003). The prevalence of sleep disturbances in studies with sample sizes <927 (65.7%, 95% CI: 36.4–89.6%) was significantly higher than that in those with sample size ≥ 927 (29.6%, 95% CI: 24.2–35.2%) (*Q* = 5.77, *p* = 0.016). The prevalence of sleep disturbances in studies with a PSQI cut-off value ≥ 7 (93.5%, 95% CI: 89.4–96.7%) was significantly higher than that in those with a PSQI cut-off value ≥ 8 (44.4%, 95% CI: 32.4–56.8%) (*Q* = 62.28, *p* < 0.001). Meta-regression analyses revealed that a smaller sample size (*B* = −0.0002, z = −2.55, *p* = 0.024) was associated with a higher sleep disturbance prevalence. Mean age (*B* = 0.02584, z = 0.85, *p* = 0.419) and study quality (*B* = −0.08162, z = −0.83, *p* = 0.420) were not associated with prevalence.

**Table 2:** Subgroup analyses of the prevalence of sleep disturbances in Chinese healthcare workers.

**Subgroups**	**Categories (No. of studies)**	**Events**	**Sample size**	**prevalence (%)**	**95%CI**	***I^**2**^*(%)**	***p-value***	***Q* (*p-*value)**
Instruments	PSQI (9)	1,998	6,168	58.0	43.4–71.9	99.1	<0.001	96.05 (<0.001)
	ISI (4)	1,581	4,844	32.2	25.1–39.9	96.8	<0.001	
	AIS (1)	311	948	32.8	29.8–35.9	–	–	
	Item from PSQI (1)	42	371	11.3	8.3–15.0	–	–	
Sex	Female (3)	871	2,368	36.7	34.8–38.7	0	<0.001	9.10 (0.003)
	Male (3)	167	575	28.6	24.2–33.2	0	<0.001	
Area	Wuhan (4)	618	1,334	49.4	34.2–64.7	96.20	<0.001	1.245 (0.264)
	Other regions (12)	2,234	7,106	39.3	30.9–48.1	97.94	<0.001	
Frontline/infected HWs	Frontline (7)	1,075	2,815	57.4	38.8–75.0	98.88	<0.001	96.96 (<0.001)
	Non-frontline (4)	567	1,792	40.0	29.9–50.6	93.45	<0.001	
	Infected (2)	101	108	97.0	92.5–99.7	0	<0.001	
Professional group	Doctor (3)	288	995	35.1	24.4–46.5	90.48	<0.001	0.84 (0.358)
	Nurse (6)	1,076	3,118	43.0	30.7–55.9	97.72	<0.001	
Sample size	<927 (7)	566	1,138	65.7	36.4–89.6	98.9	<0.001	5.77 (0.016)
	≥927 (8)	3,366	11,193	29.6	24.2–35.2	97.6	<0.001	
Cut-off of PSQI	≥7 (2)	173	190	93.5	89.4–96.7	98.6	<0.001	62.28 (<0.001)
	≥8 (7)	1,825	5,978	44.4	32.4–56.8	95.0	<0.001	

*HWs, healthcare workers*.

### Sleep Quality Assessed by the PSQI

**Figure 3:**
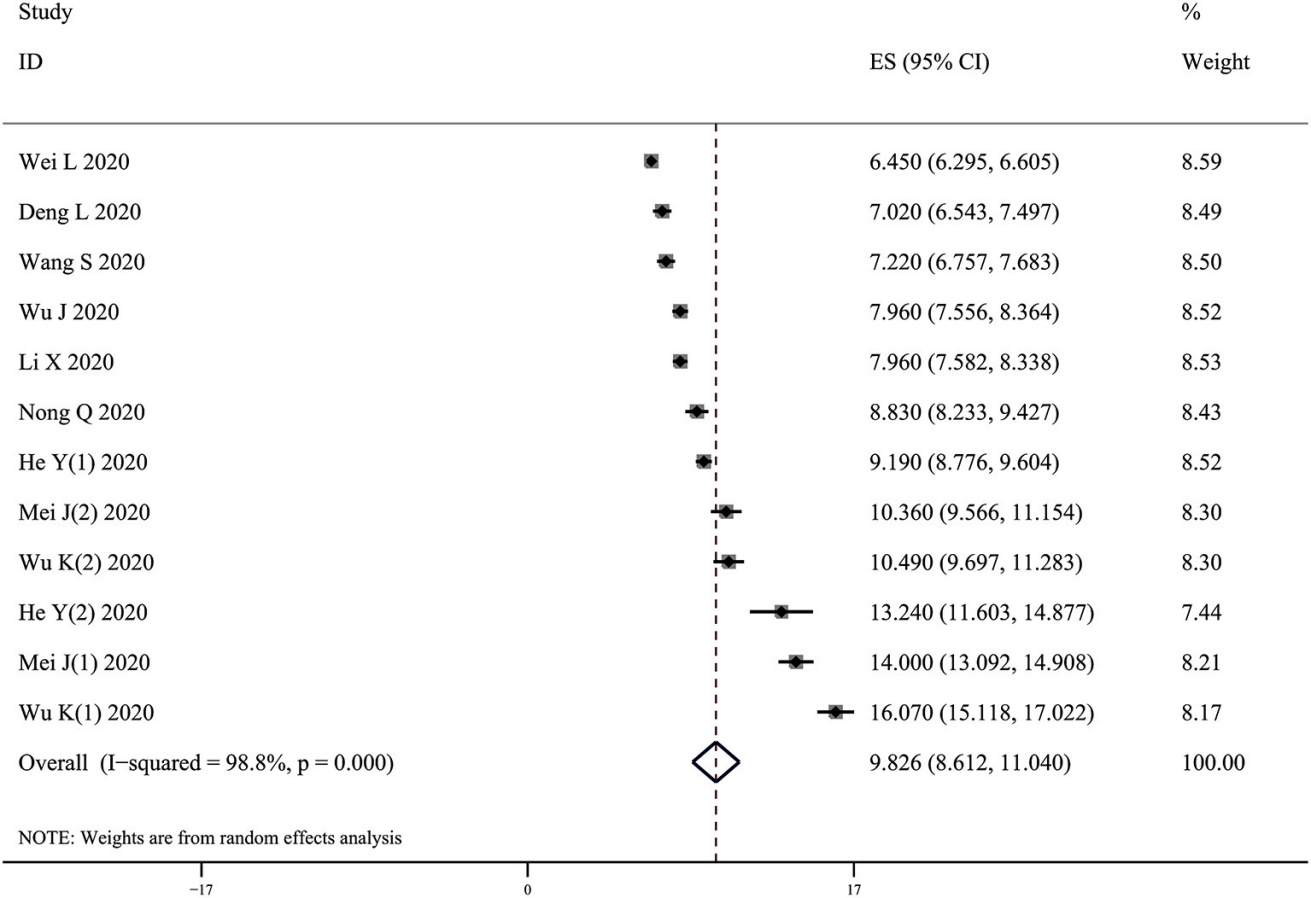
Forest plot of the mean total score of the PSQI.

The pooled mean total score of the PSQI in 9 studies with 3,338 HWs was 9.83 (95% CI: 8.61–11.04, *I*^2^ = 98.8%) (Figure 3). In the subgroup analyses, HWs from Wuhan had a higher total PSQI score (10.87, 95% CI: 8.99–12.74) than those from other regions (7.54, 95% CI: 6.50–8.59) (*Q* = 9.21, *p* = 0.002). A statistical difference in the score among frontline (9.59, 95% CI: 7.77–11.40), infected (13.82, 95% CI: 13.03–14.62), and non-frontline HWs was observed (8.84, 95% CI: 5.63–12.04) (*Q* = 24.20, *p* < 0.001) (Table 3).

**Table 3:** Subgroup analyses of the mean total score of PSQI.

**Subgroups**	**Categories (No. of studies)**	**Sample Size**	**Mean**	**SE**	**95% CI**	***I*^**2**^ (%)**	***p-*value**	***Q* (*p-*value)**
Sex	Female (3)	1,582	7.17	0.09	6.61–7.73	84.9	<0.001	0.02 (0.884)
	Male (3)	904	6.99	0.11	4.70–9.29	95.8	<0.001	
Area	Wuhan (3)	517	10.87	0.17	8.99–12.74	97.8	<0.001	9.21 (0.002)
	Other regions (4)	2,578	7.54	0.07	6.50–8.59	96.9	<0.001	
Frontline/infected HWs	Frontline (5)	559	9.59	0.14	7.77–11.40	98.5	<0.001	24.20 (<0.001)
	Non-frontline (2)	183	8.84	0.24	5.63–12.04	97.9	<0.001	
	Infected (2)	108	13.82	0.42	13.03–14.62	0	<0.001	

*HWs, healthcare workers*.

### Sensitivity Analysis and Publication Bias

**Figure 4:**
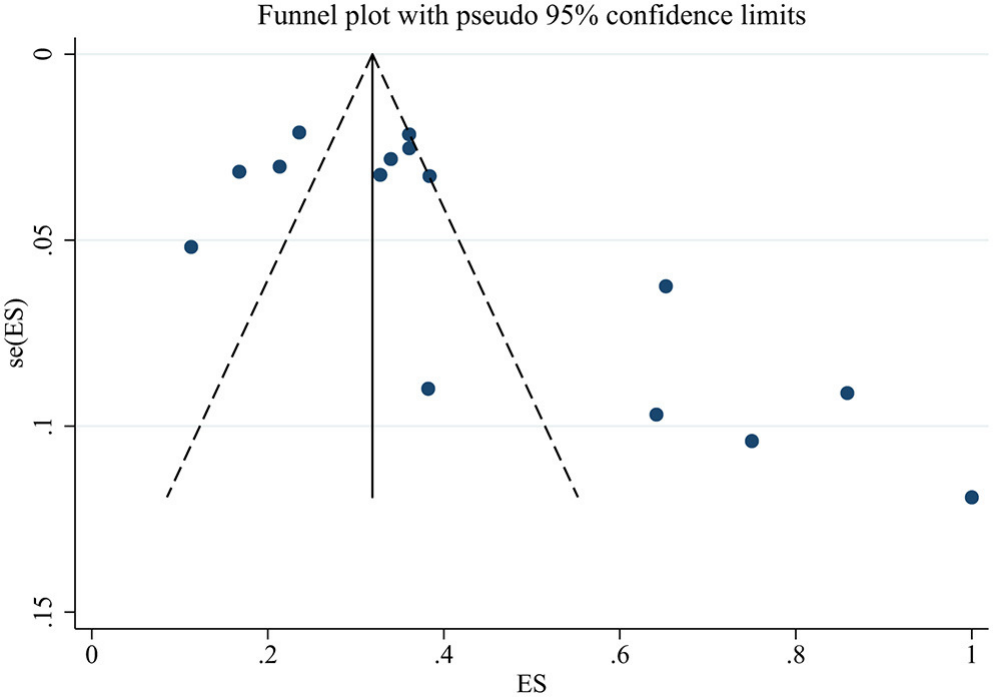
Funnel plot of publication bias for the prevalence of sleep disturbances.

The sensitivity analyses showed that no individual study significantly changed the prevalence of sleep disturbances or the mean total score of the PSQI (Supplementary Figures 1, 2). The funnel plot graphic and Egger's test (*t* = 2.74, *p* = 0.017) for the prevalence of sleep disturbances indicated some potential publication bias (Figure 4). The “trim and fill” method was performed. However, we did not identify missing studies according to the “trim and fill” method.

## Discussion

This systematic review and meta-analysis explored the pooled prevalence of sleep disturbances and sleep quality in Chinese HWs during the COVID-19 pandemic. A recent systematic review and meta-analysis examined the prevalence of sleep disturbances (34.32%, 95% CI: 27.45–41.54%) in HWs but included only five studies and did not report the results of sleep quality or data from articles published in Chinese (44). In this current meta-analysis of 18 studies with 12,736 participants, we found that the pooled prevalence of sleep disturbances in Chinese HWs during the COVID-19 pandemic was 45.1% (95% CI: 37.2–53.1%), which was approximately 1.5 times higher than that in the general population (29.2%, 95% CI: 28.8–29.6%) during the same period (45). Our finding was slightly higher than that in Chinese HWs before the COVID-19 outbreak (39.2%, 95% CI: 36.0–42.7%) (46). These findings indicate that a considerable number of Chinese HWs suffered from sleep disturbances, and the number increased during the COVID-19 pandemic.

Furthermore, we found that the prevalence of sleep disturbances in frontline and infected HWs was numerically higher than that in non-frontline HWs. During the most trying months, frontline HWs were at high risk of infection in an isolated environment, had a lack of contact with their families, and experienced frustration and discrimination. Consequently, they were prone to a series of mental health problems (47). Two recent studies found that frontline HWs were twice as likely to develop symptoms of depression and anxiety as nonclinical workers who were not in direct contact with patients with coronavirus pneumonia (48, 49). Frequent sleep disturbances in HWs may be a clinical symptom of depression and anxiety and could also contribute to their development (13–15). Infected HWs experienced more fear and loneliness than other populations, as well as a range of symptoms of infection, including fever, cough, and difficulty breathing. In addition, side effects of treatment for COVID-19, such as corticosteroids, may cause sleep disturbances.

In this study, significant differences in the prevalence of sleep disturbances in HWs among studies that used different assessment instruments were observed, consistent with the findings of previous meta-analyses including different populations (24, 25). In the nine studies using the PSQI, the prevalence was 58.0%, which was higher than that in other studies. There are some possible explanations for this discrepancy. Two studies (32, 37) used a lower PSQI cut-off value (≥7), and the study by Mei et al. (37) reported a 100% prevalence rate for sleep disturbances in infected HWs, which led to an overall higher prevalence rate in the studies using the PSQI. After excluding these two studies, the prevalence of sleep disturbances decreased to 44.4% in the subgroup analysis (Table 2). It should be noted that different methodologies in sampling and assessment across studies may affect the results to an uncertain extent. In addition, we found that a higher prevalence of sleep disturbances was associated with a smaller sample size. Studies with small sample sizes usually have unstable results, which could lead to a biased prevalence of sleep disturbances (24).

We found that female HWs were more likely to experience sleep disturbances than male HWs. The relationship between sex and sleep disturbances has been difficult to determine in different population groups. For example, in two previous studies, the gender difference in the prevalence of sleep disturbances was not statistically significant in college students (25) or elderly individuals (24). In contrast, a recent meta-analysis (46) found that female HWs had a higher prevalence than male HWs (46.4 *vs*. 33.8%, *p* < 0.001), similar to the results of this study. This finding suggests that more attention should be paid to the sleep problems in female HWs.

Sleep quality was assessed by the PSQI in 9 included studies using the mean and standard deviation of total scores. HWs in this study had a higher total PSQI score (9.83) than those in the period before the COVID-19 outbreak (5.33–7.34) (50–52), indicating that they experienced decreased sleep quality during the COVID-19 pandemic. Both the high prevalence of sleep disturbances and poor sleep quality revealed that the COVID-19 pandemic had a serious negative impact on the mental health of Chinese HWs.

The prevalence of sleep disturbances in HWs in Wuhan appeared to be higher than those in other regions (49.4 *vs*. 39.3%), although the difference was not statistically significant. Moreover, we found that compared to HWs in other regions, those in Wuhan had a higher total PSQI score (10.87 *vs*. 7.54). HWs in Wuhan were under tremendous pressure to fight COVID-19 (53). Especially in the early stage of the outbreak, there was a serious shortage of medical personnel and protective equipment in Wuhan. As a result, the mental health effects of the COVID-19 pandemic vary among different regions and periods, which requires targeted psychological interventions according to the intensity of the epidemic.

A position paper calling for the protection of HWs during the COVID-19 pandemic emphasized that in addition to supplies of personal protective equipment and food, family support and psychological support were also needed (54). To address sleep problems in HWs who had an increased work burden during the COVID-19 pandemic, the European Academy for Cognitive-Behavioral Treatment of Insomnia (the European CBT-I Academy) taskforce provided some recommendations, such as expressing stress and concerns to family members; relaxing by reading or performing yoga instead of exercising or eating before going to bed; addressing the consequences of sleep problems in a timely manner, etc. (55).

There are several strengths of this study. This meta-analysis included 17 studies obtained from both English and Chinese language databases with a considerable number of participants and revealed that a high proportion of HWs suffered from sleep disturbances during the COVID-19 pandemic. Moreover, the subgroup analyses of the pooled prevalence and sleep quality based on sex, area (Wuhan/other regions), and type of HWs (frontline/non-frontline/infected HWs) identified vulnerable groups whose mental health was more easily affected and provided a reliable indication for targeted interventions.

However, several limitations of this meta-analysis should be noted. First, most studies reported an online sampling method with a lack of details and did not clearly describe non-responders, which lowered the quality of the studies. Second, high heterogeneity was observed among studies and remained in the subgroup analyses; however, this was also difficult to avoid in previous meta-analyses of observational surveys (24, 46). The heterogeneity among studies was probably related to additional factors, such as workload, stress, and mental health, which were not included in the analyses. Third, publication bias in the prevalence of sleep disturbances may exist. However, no missing studies were found according to the “trim and fill” method. The asymmetry of funnel plots has many possible sources other than publication bias, such as poor methodological quality, true heterogeneity, artifactual, and chance (27).

## Conclusion

In summary, the prevalence of sleep disturbances was very high in Chinese HWs during the COVID-19 pandemic, particularly in frontline and infected HWs. Our results indicate a heavy mental health burden on HWs during the COVID-19 pandemic in China and provide other countries with valuable information that may help guide interventions during the crisis. Targeted interventions are urgently needed to protect the vulnerable group of HWs from mental injury and enhance their resilience.

## Data Availability Statement

The original contributions generated for this study are included in the article/Supplementary Material, further inquiries can be directed to the corresponding author/s.

## Author Contributions

LX and CC conceived and design the study. LX, ZqL, and HL performed the literature search, data extraction, and quality appraisal. XL, CG, and ZwL conducted the statistical analyses. KZ and HL provided many useful suggestions and amended the manuscript. All the authors reviewed the manuscript and approved submission to this journal.

## Conflict of Interest

The authors declare that the research was conducted in the absence of any commercial or financial relationships that could be construed as a potential conflict of interest.

## References

[1] Wang C Horby PW Hayden FG Gao GF. A novel coronavirus outbreak of global health concern. Lancet. (2020) 395:470–3. 10.1016/S0140-6736(20)30185-9 31986257 PMC7135038

[2] Huang C Wang Y Li X Ren L Zhao J Hu Y. Clinical features of patients infected with 2019 novel coronavirus in Wuhan, China. Lancet. (2020) 395:497–506. 10.1016/S0140-6736(20)30183-5 31986264 PMC7159299

[3] WHO. Coronavirus disease 2019 (COVID-19) Situation Report-132. Geneva: World Health Organization (2020). Available online at: https://www.who.int/docs/default-source/coronaviruse/situation-reports/20200531-covid-19-sitrep-132.pdf?sfvrsn=d9c2eaef_2 (accessed August 25, 2020).

[4] Beijing Daily. A Total of 3387 Medical Workers were Infected With COVID-19 in China. (2020). Available online at: https://baijiahao.baidu.com/s?id=1659420394069577730&wfr=spider&for=pc (accessed September 1, 2020).

[5] Xiang YT Jin Y Wang Y Zhang Q Zhang L Cheung T. Tribute to health workers in China: a group of respectable population during the outbreak of the COVID-19. Int J Biol Sci. (2020) 16:1739–40. 10.7150/ijbs.45135 32226292 PMC7098026

[6] National Health Commission of China. The Guidelines for Emergency Psychological Crisis Interventions for the COVID-19 Pneumonia: Bureau of Disease Control and Prevention (2020). Available online at: http://www.nhc.gov.cn/jkj/s3577/202001/6adc08b966594253b2b791be5c3b9467.shtml (accessed August 25, 2020).

[7] Nickell LA Crighton EJ Tracy CS Al-Enazy H Bolaji Y Hanjrah S. Psychosocial effects of SARS on hospital staff: survey of a large tertiary care institution. CMAJ. (2004) 170:793–8. 10.1503/cmaj.1031077 14993174 PMC343853

[8] Tam CW Pang EP Lam LC Chiu HF. Severe acute respiratory syndrome (SARS) in Hong Kong in 2003: stress and psychological impact among frontline healthcare workers. Psychol Med. (2004) 34:1197–204. 10.1017/S0033291704002247 15697046

[9] Liu Y Gayle AA Wilder-Smith A Rocklöv J. The reproductive number of COVID-19 is higher compared to SARS coronavirus. J Travel Med. (2020) 27:taaa021. 10.1093/jtm/taaa021 32052846 PMC7074654

[10] Nakata A Haratani T Takahashi M Kawakami N Arito H Kobayashi F. Job stress, social support, and prevalence of insomnia in a population of Japanese daytime workers. Soc Sci Med. (2004) 59:1719–30. 10.1016/j.socscimed.2004.02.002 15279928

[11] Yang B Wang Y Cui F Huang T Sheng P Shi T. Association between insomnia and job stress: a meta-analysis. Sleep Breath. (2018) 22:1221–31. 10.1007/s11325-018-1682-y 29959635

[12] Kucharczyk ER Morgan K Hall AP. The occupational impact of sleep quality and insomnia symptoms. Sleep Med Rev. (2012) 16:547–59. 10.1016/j.smrv.2012.01.005 22401983

[13] Taylor DJ Lichstein KL Durrence HH Reidel BW Bush AJ. Epidemiology of insomnia, depression, and anxiety. Sleep. (2005) 28:1457–64. 10.1093/sleep/28.11.1457 16335332

[14] Baglioni C Battagliese G Feige B Spiegelhalder K Nissen C Voderholzer U. Insomnia as a predictor of depression: a meta-analytic evaluation of longitudinal epidemiological studies. J Affect Disord. (2011) 135:10–9. 10.1016/j.jad.2011.01.011 21300408

[15] Li L Wu C Gan Y Qu X Lu Z. Insomnia and the risk of depression: a meta-analysis of prospective cohort studies. BMC Psychiatry. (2016) 16:375. 10.1186/s12888-016-1075-3 27816065 PMC5097837

[16] Morin CM Drake CL Harvey AG Krystal AD Manber R Riemann D. Insomnia disorder. Nat Rev Dis Primers. (2015) 1:15026. 10.1038/nrdp.2015.26 27189779

[17] Yin Q Sun Z Liu T Ni X Deng X Jia Y. Posttraumatic stress symptoms of health care workers during the corona virus disease 2019. Clin Psychol Psychother. (2020) 27:384–95. 10.1002/cpp.2477 32415733 PMC7276761

[18] Mei JH Zhang Q Gong X Li LJ Zhang Z Wang J. Analysis of psychological and sleep state of medical staff with novel coronavirus pneumonia (in Chinese). Herald Med. (2020) 39:345–9. 10.3870/j.issn.1004-0781.2020.03.017

[19] Liberati A Altman DG Tetzlaff J Mulrow C Gøtzsche PC Ioannidis JP. The PRISMA statement for reporting systematic reviews and meta-analyses of studies that evaluate healthcare interventions: explanation and elaboration. BMJ. (2009) 339:b2700. 10.1136/bmj.b2700 19622552 PMC2714672

[20] Stroup DF Berlin JA Morton SC Olkin I Williamson GD Rennie D. Meta-analysis of observational studies in epidemiology: a proposal for reporting. Meta-analysis Of Observational Studies in Epidemiology (MOOSE) group. JAMA. (2000) 283:2008–12. 10.1001/jama.283.15.2008 10789670

[21] Zhou Y Zhou Y Song Y Ren L Ng CH Xiang YT. Tackling the mental health burden of frontline healthcare staff in the COVID-19 pandemic: China's experiences. Psychol Med. (2020 13:1–2. 10.1017/S0033291720001622 32398180 PMC7417977

[22] Dong M Lu L Zhang L Zhang Q Ungvari GS Ng CH. Prevalence of suicide attempts in bipolar disorder: a systematic review and meta-analysis of observational studies. Epidemiol Psychiatr Sci. (2019) 29:e63. 10.1017/S2045796019000593 31648654 PMC8061290

[23] Yang C Zhang L Zhu P Zhu C Guo Q. The prevalence of tic disorders for children in China: a systematic review and meta-analysis. Medicine (Baltimore). (2016) 95:e4354. 10.1097/MD.0000000000004354 27472724 PMC5265861

[24] Lu L Wang SB Rao W Zhang Q Ungvari GS Ng CH. The prevalence of sleep disturbances and sleep quality in older chinese adults: a comprehensive meta-analysis. Behav Sleep Med. (2019) 17:683–97. 10.1080/15402002.2018.1469492 29851516

[25] Li L Wang YY Wang SB Zhang L Li L Xu DD. Prevalence of sleep disturbances in Chinese university students: a comprehensive meta-analysis. J Sleep Res. (2018) 27:e12648. 10.1111/jsr.12648 29383787

[26] Higgins JP Thompson SG Deeks JJ Altman DG. Measuring inconsistency in meta-analyses. BMJ. (2003) 327:557–60. 10.1136/bmj.327.7414.557 12958120 PMC192859

[27] Higgins JP Green S. Cochrane Handbook for Systematic Reviews of Interventions. Hoboken: John Wiley & Sons (2011).

[28] Egger M Davey Smith G Schneider M Minder C. Bias in meta-analysis detected by a simple, graphical test. BMJ. (1997) 315:629–34. 10.1136/bmj.315.7109.629 9310563 PMC2127453

[29] Li X Yu H Bian G Hu Z Liu X Zhou Q. Prevalence, risk factors, and clinical correlates of insomnia in volunteer and at home medical staff during the COVID-19. Brain Behav Immun. (2020) 87:140–1. 10.1016/j.bbi.2020.05.008 32380272 PMC7198418

[30] Zhang C Yang L Liu S Ma S Wang Y Cai Z. Survey of insomnia and related social psychological factors among medical staff involved in the 2019 novel coronavirus disease outbreak. Front Psychiatry. (2020) 11:306. 10.3389/fpsyt.2020.00306 32346373 PMC7171048

[31] Zhang WR Wang K Yin L Zhao WF Xue Q Peng M. Mental health and psychosocial problems of medical health workers during the COVID-19 epidemic in China. Psychother Psychosom. (2020) 89:242–50. 10.1159/000507639 32272480 PMC7206349

[32] Lai J Ma S Wang Y Cai Z Hu J Wei N. Factors associated with mental health outcomes among health care workers exposed to coronavirus disease 2019. JAMA Netw Open. (2020) 3:e203976. 10.1001/jamanetworkopen.2020.3976 32202646 PMC7090843

[33] Wang S Xie L Xu Y Yu S Yao B Xiang D. Sleep disturbances among medical workers during the outbreak of COVID-2019. Occup Med (Lond). (2020) 70:364–9. 10.1093/occmed/kqaa074 32372077 PMC7239094

[34] Huang Y Zhao N. Generalized anxiety disorder, depressive symptoms and sleep quality during COVID-19 outbreak in China: a web-based cross-sectional survey. Psychiatry Res. (2020) 288:112954. 10.1016/j.psychres.2020.112954 32325383 PMC7152913

[35] Wu K Wei X. Analysis of psychological and sleep status and exercise rehabilitation of front-line clinical staff in the fight against COVID-19 in China. Med Sci Monit Basic Res. (2020) 26:e924085. 10.12659/MSMBR.924085 32389999 PMC7241216

[36] Deng LX Dong LJ Guo XM Chen JY. Investigation on the sleep quality of front-line nurses in fight against COVID-19 and related influencing factors (in Chinese). Nurs Integr Tradit Chin West Med. (2020) 6:33–7. 10.11997/nitcwm.202003059

[37] He YH Wang Y Wang JS Hou JB Wan X. Impact of COVID-19 outbreak on sleep quality of medical staff in Wuhan (in Chinese). Med J Wuhan Univ. (2020) 41:711–14. 10.14188/j.1671-8852.2020.0159

[38] Liu XL Cheng YS Wang MY Pan Y Guo H Jiang RH. Psychological state of nursing staff in a large scale of general hospital during COVID-19 epidemic (in Chinese). Chin J Nosocomiol. (2020) 30:1641–6. 10.11816/cn.ni.2020-200572

[39] Nong QX Mo LL Zhou FZ Song L Chen J Liao HC. Investigation and analysis on mental health status and sleep quality of nurses in psychiatric hospital during COVID-19 epidemic (in Chinese). Intern Med. (2020) 15:84–6. 10.16121/j.cnki.cn45-1347/r.2020.01.28

[40] Wei L Shi LP Cao J. Psychological status of primary care workers during the COVID-19 epidemic in Shanghai (in Chinese). J Tongji Univ. (2020) 41:155–60. 10.16118/j.1008-0392.2020.02.003

[41] Wu JJ Rong X Chen F Diao YJ Chen DC Jing XC. Investigation on sleep quality of first-line nurses in fighting against corona virus disease 2019 and its influencing factors (in Chinese). Chin Nurs Res. (2020) 34:558–62. 10.12102/j.issn.1009-6493.2020.04.107

[42] Li X Lei Y Hu DY Deng XF. Construction of a three-level psychological crisis intervention system for first-line nurses in COVID-19 designated hospitals (in Chinese). J Nurses Train. (2020) 35:1015–8. 10.16821/j.cnki.hsjx.2020.25.021

[43] China Daily. Wuhan Reopens After 76-day Lockdown. (2020). Available online at: http://en.people.cn/n3/2020/0408/c90000-9677059.html (accessed January 15, 2021).

[44] Pappa S Ntella V Giannakas T Giannakoulis VG Papoutsi E Katsaounou P. Prevalence of depression, anxiety, and insomnia among healthcare workers during the COVID-19 pandemic: a systematic review and meta-analysis. Brain Behav Immun. (2020) 88:901–7. 10.1016/j.bbi.2020.11.023 32437915 PMC7206431

[45] Shi L Lu ZA Que JY Huang XL Liu L Ran MS. Prevalence of and risk factors associated with mental health symptoms among the general population in China during the coronavirus disease 2019 pandemic. JAMA Netw Open. (2020) 3:e2014053. 10.1001/jamanetworkopen.2020.14053 32609353 PMC7330717

[46] Qiu D Yu Y Li RQ Li YL Xiao SY. Prevalence of sleep disturbances in Chinese healthcare professionals: a systematic review and meta-analysis. Sleep Med. (2020) 67:258–66. 10.1016/j.sleep.2019.01.047 31040078

[47] Greenberg N Docherty M Gnanapragasam S Wessely S. Managing mental health challenges faced by healthcare workers during covid-19 pandemic. BMJ. (2020) 368:m1211. 10.1136/bmj.m1211 32217624

[48] Liu CY Yang YZ Zhang XM Xu X Dou QL Zhang WW. The prevalence and influencing factors in anxiety in medical workers fighting COVID-19 in China: a cross-sectional survey. Epidemiol Infect. (2020) 148:e98. 10.1017/S0950268820001107 32430088 PMC7251286

[49] Lu W Wang H Lin Y Li L. Psychological status of medical workforce during the COVID-19 pandemic: a cross-sectional study. Psychiatry Res. (2020) 288:112936. 10.1016/j.psychres.2020.112936 32276196 PMC7195354

[50] Dong H Zhang Q Sun Z Sang F Xu Y. Sleep problems among Chinese clinical nurses working in general hospitals. Occup Med. (2017) 67:534–9. 10.1093/occmed/kqx124 29016953

[51] Chien PL Su HF Hsieh PC Siao RY Ling PY Jou HJ. Sleep quality among female hospital staff nurses. Sleep Disord. (2013) 2013:283490. 10.1155/2013/283490 23766916 PMC3666224

[52] Yang XM Wang RF Liu ZQ Wang Q. Relationship between sleep quality and mental health of physicians (in Chinese). China J Health Psychol. (2007) 15:654–6. 10.13342/j.cnki.cjhp.2007.07.040

[53] Kang L Li Y Hu S Chen M Yang C Yang BX. The mental health of medical workers in Wuhan, China dealing with the 2019 novel coronavirus. Lancet Psychiatry. (2020) 7:e14. 10.1016/S2215-0366(20)30047-X 32035030 PMC7129673

[54] The Lancet. COVID-19: protecting health-care workers. Lancet. (2020) 395:922. 10.1016/S0140-6736(20)30644-9 PMC7138074 32199474

[55] Altena E Baglioni C Espie CA Ellis J Gavriloff D Holzinger B. Dealing with sleep problems during home confinement due to the COVID-19 outbreak: practical recommendations from a task force of the European CBT-I Academy. J Sleep Res. (2020) 29:e13052. 10.1111/jsr.13052 32246787

