# Supplementary material for: Prevalence of Sleep Disturbances and Sleep Quality in Chinese Healthcare Workers During the COVID-19 Pandemic: A Systematic Review and Meta-Analysis
Source: Front Psychiatry. 2021 Feb 18;12:646342. doi: 10.3389/fpsyt.2021.646342 (PMC7930820; doi:10.3389/fpsyt.2021.646342)

Supplementary Table 1. Quality assessment of the studies included in the meta-analysis

| NO. | Study | 1. Is the target population clearly defined? | 2. Was either of the following ascertainment methods used [must be one or the other]?(1) probability sampling, or (2) entire population surveyed | 3. Is the response rate >70% | 4. Are non-responders clearly described? | 5. Is the sample representative of the target population? | 6. Were data collection methods standardized? | 7. Were validated criteria used to assess for the presence/absence of disease? | 8. Are the estimates of prevalence given with confidence intervals and in detail by subgroup (if applicable)? | Total score |
| --- | --- | --- | --- | --- | --- | --- | --- | --- | --- | --- |
| 1 | Li X 2020 | 1 | 0 | 0 | 0 | 1 | 1 | 1 | 1 | 5 |
| 2 | Zhou Y 2020 | 1 | 0 | 0 | 0 | 1 | 1 | 1 | 1 | 5 |
| 3 | Zhang C 2020 | 1 | 0 | 0 | 0 | 1 | 1 | 1 | 0 | 4 |
| 4 | Zhang W 2020 | 1 | 0 | 0 | 0 | 1 | 1 | 1 | 0 | 4 |
| 5 | Lai J 2020 | 1 | 1 | 0 | 1 | 1 | 1 | 1 | 0 | 6 |
| 6 | Yin Q 2020 | 1 | 0 | 1 | 0 | 1 | 1 | 1 | 0 | 5 |
| 7 | Wang S 2020 | 1 | 0 | 0 | 0 | 1 | 1 | 1 | 0 | 4 |
| 8 | Huang Y 2020 | 1 | 0 | 1 | 0 | 1 | 1 | 1 | 0 | 5 |
| 9 | Wu K 2020 | 1 | 0 | 0 | 0 | 1 | 1 | 1 | 0 | 5 |
| 10 | Deng L 2020 | 1 | 0 | 1 | 0 | 1 | 1 | 1 | 0 | 5 |
| 11 | He Y 2020 | 1 | 0 | 0 | 0 | 1 | 1 | 1 | 0 | 4 |
| 12 | Liu X 2020 | 1 | 0 | 1 | 0 | 1 | 1 | 1 | 0 | 5 |
| 13 | Mei J 2020 | 1 | 0 | 0 | 0 | 1 | 1 | 1 | 0 | 4 |
| 14 | Nong Q 2020 | 1 | 1 | 0 | 0 | 1 | 1 | 1 | 0 | 5 |
| 15 | Wei L 2020 | 1 | 1 | 1 | 0 | 1 | 1 | 1 | 0 | 6 |
| 16 | Wu J 2020 | 1 | 0 | 1 | 0 | 1 | 1 | 1 | 0 | 5 |
| 17 | Li X 2020 | 1 | 0 | 0 | 0 | 1 | 1 | 1 | 0 | 4 |

Supplementary Figure 1. Sensitivity analysis of the prevalence of sleep disturbances


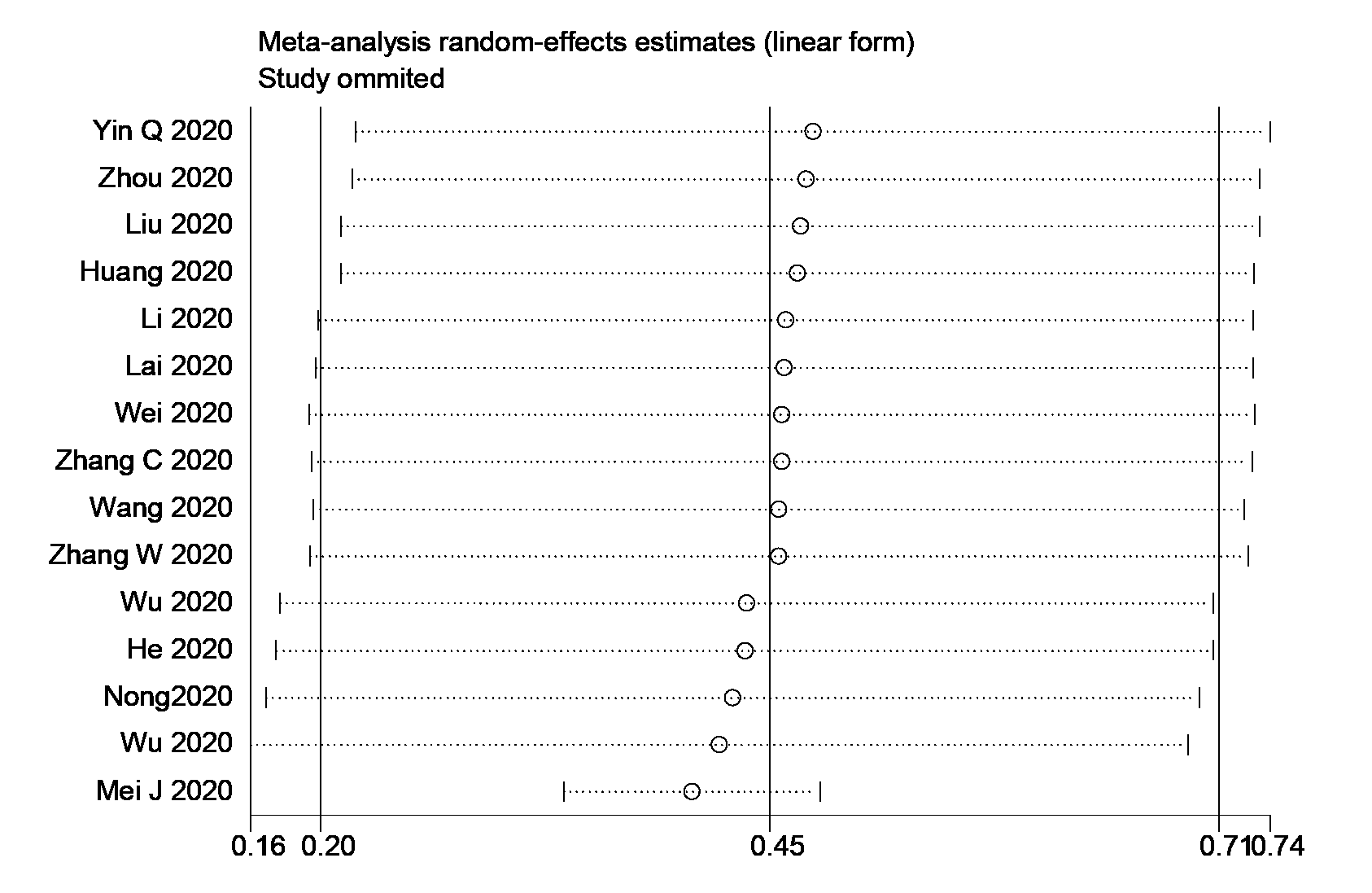


Supplementary Figure 2. Sensitivity analysis of the mean total score of PSQI


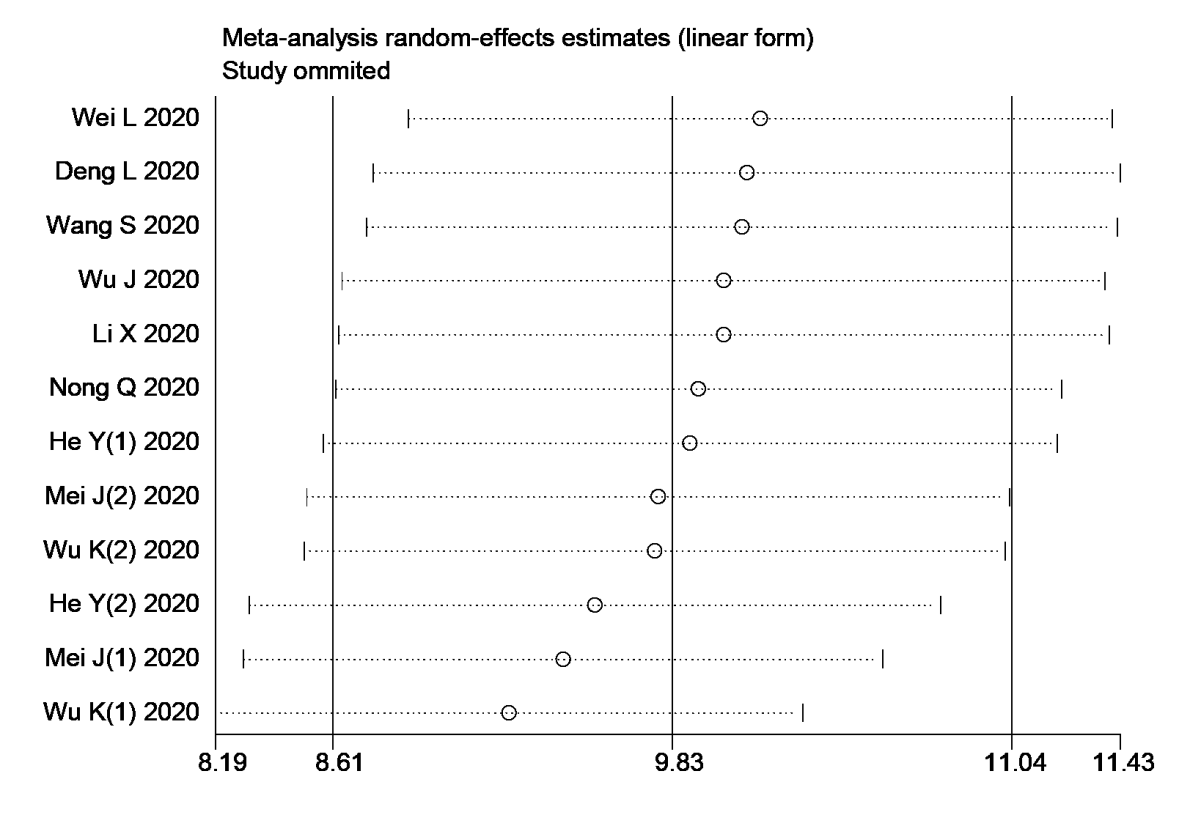

Supplement: Supplementary file 1 [file Data_Sheet_1.docx]
